# Major and ancillary magnetic resonance features of LI-RADS to assess HCC: an overview and update

**DOI:** 10.1186/s13027-017-0132-y

**Published:** 2017-04-28

**Authors:** Vincenza Granata, Roberta Fusco, Antonio Avallone, Orlando Catalano, Francesco Filice, Maddalena Leongito, Raffaele Palaia, Francesco Izzo, Antonella Petrillo

**Affiliations:** 1 Radiology Division, “Istituto Nazionale Tumori - IRCCS - Fondazione G. Pascale”, Via Mariano Semmola, Naples, Italy; 2 Abdominal Oncology Division, “Istituto Nazionale Tumori - IRCCS - Fondazione G. Pascale”, Via Mariano Semmola, Naples, Italy; 3 Hepatobiliary Surgery Division, “Istituto Nazionale Tumori - IRCCS - Fondazione G. Pascale”, Via Mariano Semmola, Naples, Italy

**Keywords:** HCC, LI-RADS, Magnetic resonance imaging

## Abstract

Liver Imaging Reporting and Data System (LI-RADS) is a system for interpreting and reporting of imaging features on multidetector computed tomography (MDCT) and magnetic resonance (MR) studies in patients at risk for hepatocellular carcinoma (HCC). American College of Radiology (ACR) sustained the spread of LI-RADS to homogenizing the interpreting and reporting data of HCC patients. Diagnosis of HCC is due to the presence of major imaging features. Major features are imaging data used to categorize LI-RADS-3, LI-RADS-4, and LI-RADS-5 and include arterial-phase hyperenhancement, tumor diameter, washout appearance, capsule appearance and threshold growth. Ancillary are features that can be used to modify the LI-RADS classification. Ancillary features supporting malignancy (diffusion restriction, moderate T2 hyperintensity, T1 hypointensity on hapatospecifc phase) can be used to upgrade category by one or more categories, but not beyond LI-RADS-4. Our purpose is reporting an overview and update of major and ancillary MR imaging features in assessment of HCC.

## Background

Hepatocellular carcinoma (HCC) is one of the most common human solid malignancies worldwide [1, 2]. The most important risk factor for the development of HCC is liver cirrhosis, regardless of its etiology [1]. Among patients with cirrhosis, those with chronic viral infection (hepatitis B and C) and high alcohol intake have the highest risks of HCC development. Imaging surveillance is a widely accepted tool that increases the likelihood of early detection of HCC and an accurate detection and characterization of focal liver nodule on patient at risk for HCC is mandatory since the management of HCC patients differs to other malignant or benign nodules [2]. According to National Comprehensive Cancer Network (NCCN) [3] and the guidelines of European Association for the Study of the Liver (EASL) and American Association for the Study Liver Diseases (AASLD), diagnostic criteria, to characterize HCC, can only be applied to cirrhotic patients and should be based on the detection of the typical hallmark of HCC (hypervascular in the arterial phase with washout in the portal venous or delayed phases) [4]. However, the current imaging-based criteria have several limitations, including the lack of established consensus regarding the exact definitions of imaging features, binary categorization (either definite or not definite HCC), and failure to address non-HCC malignancies and vascular invasion [5]. Therefore American College of Radiology (ACR) sustained the spread of Liver Imaging Reporting and Data System (LI-RADS) to homogenizing the interpreting, reporting and data collection of HCC imaging [6]. LI-RADS is a scheme for interpreting and reporting of imaging features on multidetector computed tomography (CT) and magnetic resonance (MR) studies in patients at risk for hepatocellular carcinoma (HCC) [5–7]. In the current (v. 2014) LI-RADS [6], the diagnosis of HCC is based on the presence of major imaging features. These are features used to categorize LI-RADS- category 3 (LR-3), LI-RADS- category 4 (LR-4), and LI-RADS- category 5 (LR-5) and include arterial-phase hyperenhancement, tumor diameter, washout appearance, capsule appearance, and threshold growth [6]. Ancillary features are imaging features that can be used to change the LI-RADS category [5]. Ancillary features favoring malignancy (diffusion restriction, moderate T2 hyperintensity, T1 hypointensity on hepatospecific phase) can upgrade category, but not beyond LR-4. In contrast, ancillary features favoring benignity can decrease category [5, 6].

As required in most clinical trials, MDCT presents the key imaging modality in the patient assessment. This is due to its wide availability, standardization, and ability to scan the whole abdomen and chest in one setting. MRI plays a role in HCC assessment of patients with contraindication to iodine contrast medium [8]. However, considering the evidences on the accuracy of the various imaging modalities on HCC assessment [9], so as the guidelines of the European Society of Gastrointestinal and Abdominal Radiology (ESGAR) Working Group [10], MRI is the technique to choose in pre-treatment setting. It is a valuable diagnostic tool providing lesion morphological and functional data, thanks to hepatospecific contrast medium and DW sequences [11–14].

To standardize imaging technique among institutions, LI-RADS outlines technical requirements for MRI. Precontrast, arterial phase, portal venous phase, and delayed phase are all required for MRI with extracellular agents. Each phase contributes to characterization of LI-RADS major features. For MRI with hepatobiliary agents, a delay of 15–20 min for gadoxetic acid and a delay of 1 h for gadobenate dimeglumine consistently provide high-quality hepatobiliary phase imaging. In the setting of cirrhosis increasing the delay for hepatobiliary phase imaging to 30 min or more for gadoxetic acid and 2–3 h for gadobenate dimeglumine may improve parenchymal enhancement somewhat [6]. Although the delayed phase cannot be used to evaluate washout appearance, it can be used to evaluate capsule appearance, a major feature of HCC. Also, the delayed phase and hepatobiliary phase can be used to evaluate hypointensity on both sequences; these are ancillary features favoring malignancy and so can be used to upgrade the category. Late arterial phase is strongly preferred over early arterial phase, as HCC enhancement usually is greater in the late than in the early phase, and some HCCs show hyperenhancement only in the late arterial phase [6]. Unenhanced T1-weighted (T1-W) out of phase (OP)/in phase (IP) is required. T1-W OP/IP allows identification of fat and iron and is necessary for assessment of some ancillary features. T2-W sequences are required, improving distinction between solid and nonsolid lesions and are necessary for assessment of some ancillary LI-RADS features. DWI is suggested but not required [6].

Our purpose is reporting an overview and update of major and ancillary MR imaging features in assessment of HCC.

## Methods

This overview and update is the result of autonomous studies without protocol and registration number.

### Search criterion

Several electronic dataset were searched: PubMed (US National Library of Medicine, http://www.ncbi.nlm.nih.gov/pubmed), Scopus (Elsevier, http://www.scopus.com/), Web of Science (Thomson Reuters, http://apps.webofknowledge.com/) and Google Scholar (https://scholar.google.it/). The following search criteria have been used: “hepatocellular carcinoma” AND “diffusion magnetic resonance imaging” AND “characterization, “hepatocellular carcinoma” AND “dynamic contrast enhanced magnetic resonance imaging” AND “characterization, “hepatocellular carcinoma” AND “EOB-GD-DTPA contrast medium” AND “characterization, “hepatocellular carcinoma” AND “multimodal imaging” AND “characterization”. The search covered the years from January 2000 to January 2017. Moreover, the reference lists of the found papers were analysed for papers not indexed in the electronic databases.

All titles and abstracts were analysed and exclusively the studies reporting MRI, EOB-GD-DTPA MRI, DWI results in the characterization of HCC were retained.

The inclusion criteria were: clinical study evaluating MR assessment of HCC, clinical study evaluating functional MR imaging criteria in the assessment of patients with HCC, and clinical study evaluating DWI and EOB-GD-DTPA to assessing HCC patient. Articles published in the English language from January 2000 to January 2017 were included. Exclusion criteria were unavailability of full text, general overview articles and congress abstracts; studies with lesion higher than 20 mm. There was not define a minimum number of patients as an inclusion criteria.

## Results

**Fig. 1:**
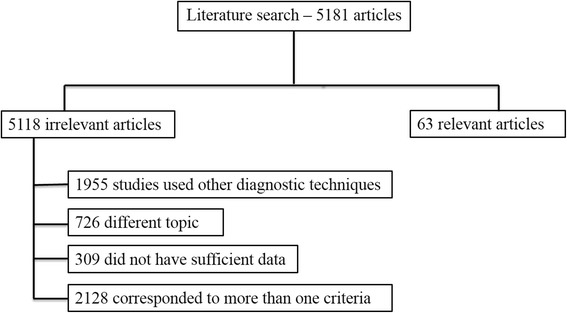
Included and excluded studies in systematic review

By using the search terms described earlier, we identified 5181 studies from January 2000 to January 2017. To identify additional relevant studies, the reference lists of the retrieved studies were checked manually. 1955 studies used other diagnostic techniques than MRI, EOB-GD-DTPA-MRI and DWI, 726 have different topic respect to characterization; 309 did not have sufficient data (case report, review, letter to editors); 2128 corresponded to more than one criteria so 63 articles were included at the end (Fig. 1).

## Discussion

Early diagnosis is a critical step in the management of HCC patients. The identification of the specific vascular profile characterized by contrast arterial uptake followed by washout in the venous phases has allowed defining the non-invasive diagnostic criteria for HCC according to AASLD and EASL-EORTC guidelines [4, 5]. The typical hallmark has 100% specificity when demonstrated on dynamic contrast study, both on CT than on MRI, in patients at high risk of HCC [1]. However, arterial hyperehnancement and wash out appearance have a sensitivity rate of 50–60% in lesion smaller than 2 cm and thus a biopsy is still needed [15]. The typical vascular profile is correlated to hemodynamic changes in nodule during hepatocarcinogenesis, and to understand the hemodynamics of HCC is important for the accurate diagnostic analysis, because there is an intense correlation between their hemodynamics and pathophysiology [16]. Angiogenesis such as sinusoidal capillarization and unpaired arteries shows gradual increase during carcinogenesis from high-grade dysplastic nodule to classic hypervascular HCC. In accordance with this angiogenesis, the intranodular portal supply is decreased, whereas the intranodular arterial supply is first decreased during the early stage and then increased in parallel with increasing grade of malignancy of the lesion. On the other hand, the main drainage vessels of hepatocellular nodules change from hepatic veins to hepatic sinusoids and then to portal veins, mainly due to disappearance of the hepatic veins from the nodules [16]. The nodule appearance on arterial phase relative, considering the intra-lesion arterial supply, can be categorized into four types. Type I when the nodule is isodense to the surrounding cirrhotic liver parenchyma, and it is due to the same intranodular arterial blood supply relative to the surrounding liver. Type II, when the nodule is hypodense to the surrounding cirrhotic liver parenchyma, indicating decreased arterial blood supply. Type III a part of the nodule demonstrating hyperdensity due a partially increased arterial supply and type IV entirely hyperdense indicating entirely increased arterial supply [16, 17]. These findings reveal the significant correlation or strong tendency between type I and low grade dysplastic nodule and early HCC, type II and high grade dysplastic nodule and early HCC, type III and well differentiated HCC and type IV and moderately or poorly differentiated HCC [16, 17]. Also in early HCC, there is not perinodular enhancement on portal or equilibrium phase of contrast study, but it is definite in hypervascular classical HCC.

During hepatocarcinogenesis multi-step changes of drainage vessels and peritumoral enhancement occurred. In dysplastic nodules or early HCCs, the main drainage route from the tumor is intranodular or perinodular hepatic vein. However, because hepatic veins disappear from the tumor during very early stage of hepatocarcinogenesis, drainage vessels change to hepatic sinusoids. This drainage was well visualized in the late phase of contrast studies. Histological examination revealed continuity between a tumor sinusoid and a portal venule in the pseudocapsule (encapsulated HCC) or surrounding hepatic sinusoids (HCC without pseudocapsule). In moderately differentiated HCC with pseudocapsule formation, the communication between tumor sinusoids and the surrounding hepatic sinusoids are also blocked, and then, the portal venules in the pseudo-capsule finally become the main drainage vessel from the tumor. In accordance with the changes of the drainage vessels, thin to thick corona enhancement appears surrounding the tumor. Corona enhancement is thicker in encapsulated HCC and thin in HCC without pseudocapsule [16].

### Arterial phase hyperenhancement

Arterial phase hyperenhancement is an essential prerequisite for definitely HCC (LR-5), but it is non-specific. In fact considering the hepatocarcinogenesis this feature may be not present, so as it may be observed in benign entities such as dysplastic nodules and arterio-portal shunts [1, 2]. Holland et al. showed, in proven HCC patients, that the majority (93%) of hypervascular lesions on arterial phase that were not detected on T2-W and portal and/or equilibrium phase of contrast study were non-neoplastic [18]. Conversely, Kim et coworkers [19] demonstrated that the most significant findings associated with HCC, in nodules smaller than 20 mm, were arterial phase hypernhancement. Ehman et al. demonstrated that arterial hypenhancement was the most commonly observed major criterion on 159 (86%) of 184 proven HCC, and was seen slightly more frequently at CT vs. MRI (87 vs. 86%, *p* = 1.00). Between the two readers, there was agreement on arterial phase characteristics in 156 (95%) cases (κ = 0.75) [20]. Conversely Burrel et al. [21] showed that sensitivity of MR was superior to CT to detect HCC (58/76 [76%] versus 43/70 [61%], respectively). Sensitivity of MR for detection of additional nodules decreased with size (>20 mm: 6/6 [100%]; 10–20 mm: 16/19 [84%]; <10 mm: 7/22 [32%]) and was superior to CT for nodules 10 to 20 mm (84 vs. 47%). Non specific hypervascular nodules >5 mm at MR were HCC in two thirds of the cases [21]. Special attention must be given to perfusion alterations, common condition in cirrhotic livers that may be false positive. These are areas of arterial hyperenhancement most frequently caused by arterioportal shunts [22, 23]. These alterations are usually peripheral, wedge shaped, and isointense relative to the surrounding parenchyma on T1- and T2-W MR images, and can be confidently characterized as LR-1. Perfusion alterations can also be nodular and it is difficult to distinguish from a true lesion [18, 23]. Areas of nodular arterial hyperenhancement seen exclusively during the arterial phase are more appropriately categorized as LR-2 [13, 18], but if corresponding others observations (eg, hyperintensity T2 signal or restricted diffusion) should be categorized as either LR-3 or LR-4 depending on its size and nonvascular features. Some areas of perfusion alteration can occur secondary to focal liver lesions, including HCC [24].

**Fig. 2:**
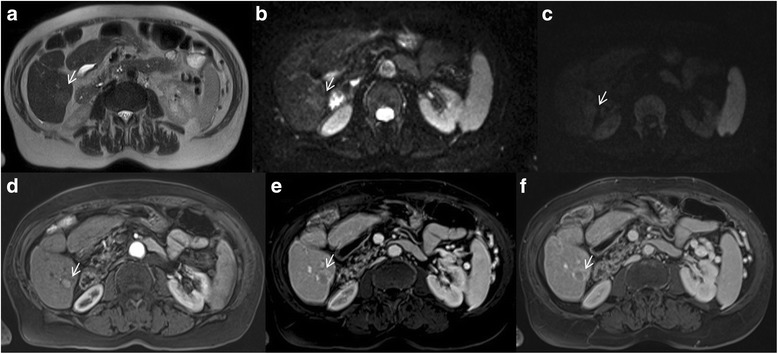
Man 73 years old with typical HCC on VI hepatic segment. The HCC is hyperintense (*arrow*) on T2-W sequences (**a**), shows (*arrows*) restrict diffusion (**b**: b50 s/mm^2^, **c**: b800 s/mm^2^). After contrast medium injection, the nodule is hypervascular (*arrow*) on arterial phase (**d**), with wash-out appearance (*arrow*) on portal phase (**e**) and capsule appearance (*arrow*) on equilibrium phase (**f**) of contrast study with Gd-BT-DO3A

Arterial hyperenhancement is the most considerable feature in patients with HCC (Fig. 2) and is considered to be the most important feature for imaging diagnosis [25–27]. This feature reflects the neoangiogenesis, which is associated with the stepwise process of carcinogenesis and becomes the dominant blood supply in overt HCC lesions [16, 17].

### Washout appearance

**Fig. 3:**
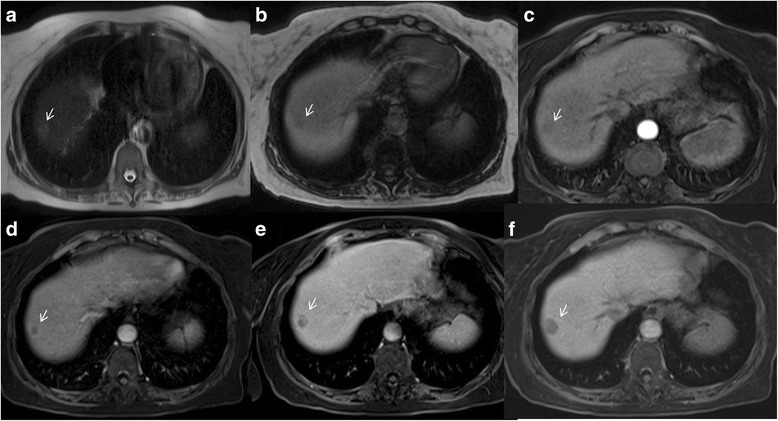
Woman 73 years old with atypical HCC on VII-VIII hepatic segment. The HCC is hyperintense (*arrow*) on T2-W sequences (**a**) and hypointense (*arrow*) on T1-W sequences (**b**: out-of-phase). During arterial phase (**c**), it is not hypervascular (*arrow*), while there is wash-out appearance (*arrow*) and capsule appearance (*arrow*) on portal phase (**d**), on equilibrium phase (**e**) and hepatospecific phase (**f**) of contrast study

Washout appearance is a reduction in contrast-enhancement relative to liver from an earlier to a later phase resulting in hypoenhancement in portal or delayed phase [28]. This may reflect multiple concomitant phenomena: rapid venous drainage, reduced portal venous supply and later enhancement of the background liver especially with hepatobiliary agents [28]. Jang et al. reported a variation in the timing of washout in the portal venous and delayed phases [29]. He reported, in a pilot study on enhancement of 112 histologically proven HCCs, that arterial phase hyperenhancement was present on 74 (77, 96%) and portal washout within 90 s on 72 (74, 97%) in the majority of moderately differentiated HCC. However, the authors found that well differentiated and poorly differentiated HCCs had an atypical enhancement patterns where 25 out of 97 (26%) showed washout between 91 and 180 s and 21 out of 97 (22%) showed late washout between 180 and 300 s [29]. Choi et al. [30] demonstrated as HCCs smaller than 1.5 cm showed typical features less frequently than HCCs 1.5 cm or larger in diameter. In subgroup analyses, HCCs with diameters between 1 and 1.5 cm showed similar MRI findings to HCCs with diameters 1 cm or less but significantly different findings compared with HCCs with diameters from 1.5 to 2 cm and 2–3 cm [31]. Portal or later hypoenhancement is considered a strong predictor of HCC, particularly when combined with arterial phase hyperenhancement [30]. Conversely Granito et al. [32] demonstrated that the most interesting result of their study was the finding of 8 HCC nodules, seven of which lacked the typical vascular pattern, which appeared as hypointense nodules on hepatobiliary phase with wash- out on portal phase, not preceded by arterial hyperenhancement (Fig. 3) [32]. This radiological pattern (wash-out and hypointense signal on hepatobiliary phase) could correspond to an early stage of carcinogenesis characterized by a reduction in both portal venous and arterial supplies [32]. The presence of wash out is a crucial step on LI-RADS decision tree for LR- 3–5 observations [1–3]. Becker et al. showed as the diameter and washout criteria using a step wise LI-RADS decision tree for LR- 3–5 observations allowed faster categorization with better inter-observer reliability while maintaining the excellent diagnostic accuracy of the most recent LI-RADS v2014 [33]. Fibrotic tissue in cirrhotic hepatic parenchyma typically shows hypointensity signal on portal or delayed phase of contrast study that may cause a false appearance of hypoenhancement of a regenerative nodule when these are surrounded by fibrosis. In some cases, fibrotic tissue may even mimic a delayed enhancing capsule or pseudocapsule [34].

### Capsule appearance

Capsule appearance is defined as a peripheral rim of smooth hyperenhancement in the portal or delayed phase (Fig. 3). The rim of enhancement is not always a true tumor capsule, but may represent a pseudocapsule corresponding to fibrous tissue and dilated sinusoids around a nodule [16, 17, 28]. Anis and coworkers showed as the capsule appearance has a high positive predictive value for HCC in at-risk patients [35]. Dioguardi Burgio et al. [36] showed as hyperintense capsule was present either on portal phase in 11/46 and in 24/25 HCCs imaged with gadoxetic acid and gadobenate dimeglumine-enhanced MR imaging, respectively (24 vs. 96%). A hypointense capsule appearance was present on hepatobiliary phase in 8/46 and 0/22 HCCs evaluated with gadoxetic acid and gadobenate dimeglumine-enhanced MR imaging, respectively (17 vs. 0%) [36]. Conversely to Dioguardi Burgio et al. that analyzed two different contrast media, Zhang et al. [37] compared diagnostic accuracy of CT and MRI to predicting of malignancy and showed that CT against MR produced false-negative findings of pseudo-capsule by 42.9% with an underestimated LI-RADS score by 16.9% for LR- 3, 37.3% for LR- 4, and 8.5% for LR- 5. CT produced significantly lower accuracy (54.3 versus 67.8%) and sensitivity (31.6 versus 71.1%) than MRI in the prediction of malignancy [37]. Also Corwin et al. [38] compared the diagnostic accuracy of CT respected to MR to grading LI-RADS. The most important finding of this study was that nearly half (42%) of observations were significantly upgraded on MRI compared with CT, and approximately one third of upgrades were to category 4, 5, or 5 V. The most common reason for the upgrade by MRI was the visualization of arterial hyperenhancement or a delayed enhancing capsule not seen on CT [38]. It is clear that these features should be correctly identified since they are major features on LI-RADS.

## Hypointense signal on hepatobiliary phase

**Fig. 4:**
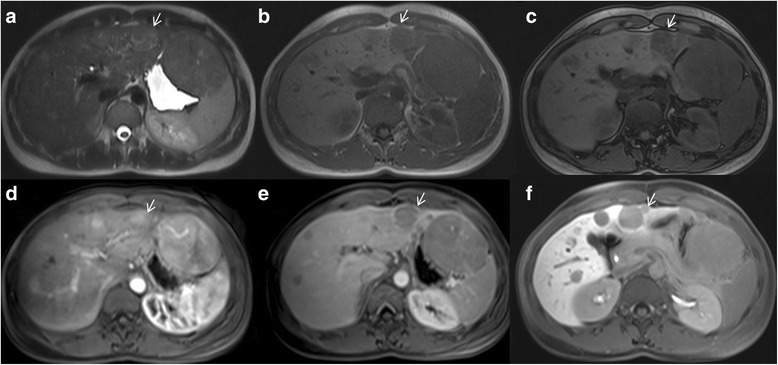
Woman 44 years old with multiple nodules of HCC. The nodules are hyperintense (*arrow*) on T2-W sequences (**a**), hypointense (*arrow*) on T1-W sequences (**b**: in-of-phase; **c**: out-of-phase), hypervascular (*arrow*) on arterial phase (**d**), with wash-out and capsule appearance (*arrow*) on portal phase (**e**) and hypointense signal (*arrow*) on hepatospecific phase (**f**) of contrast study with EOB-GD-DTPA

Hepatobiliary contrast agents are widely used in the evaluation of patients at high risk for HCC. GD-EOB-DTPA is a liver-specific agent, taken up by hepatocytes. It can be injected as an intravenous bolus, providing data about lesion vascularity in the different phases of contrast circulation. Additionally functional data can be obtained in the delayed, hepatobiliary phase [39]. Recently, the LI-RADS system incorporated the hepatobiliary phase appearance of observations as an ancillary feature that may be used to favor malignancy or benignity [40, 41]. Two ancillary features favoring malignancy on hepatobiliary phase include observation hypointensity, which is defined as the intensity of an observation lesser than the surrounding liver parenchyma, and hypointense rim, which is thought to correlate to a capsule (Fig. 4) [40, 41]. Conversely, iso-intensity of an observation to background liver favors benignity [41]; however, the study must have an adequate hepatobiliary phase, defined by LI-RADS as liver parenchyma being unequivocally hyperintense to the intrahepatic vessels. It has been demonstrated that the use of Gd-EOB-DTPA improves detection of HCC with higher sensitivity and specificity when compared to the studies with extracellular agents [40, 41]. Despites the advantages, there are also several limits. Hepatobiliary agents cost more than traditional extracellular contrast agents. Hepatobiliary agents have been associated with acute transient dyspnoea, independent of other patient risk factors [42, 43]. This dyspnoea occurs during the arterial phase imaging, therefore, degrading the study and limiting the evaluation for hepatic arterial hyperenhancement that is typical of HCC [42, 43]. Also the uptake of contrast medium by hepatocytes depends on function and the presence of membrane transporters, which are downregulated in the setting of cirrhosis [44–46]. Therefore, the utility of these agents may be clarified in patients with hepatic dysfunction. There have been several studies examining correlation of liver enhancement during the hepatobiliary phase with Child Pugh class, Model for End-stage Liver Disease (MELD) score, and various laboratory factors [47, 48]; however, no definitive cut-off values have been established for clinical parameters. As a suboptimal hepatobiliary phase would negate the advantage of these agents respect to extracellular agents [49]. Despite these limits several researches have demonstrated that EOB-GD-DTPA can favor the detection and the characterization of HCC nodule [50–56]. According to Golfieri et al. [53], during the hepatospecific phase, typical HCC and early HCC appear hypointense, whereas low-grade dysplastic or regenerative nodules appear as iso- or hyperintense lesions. The diagnostic accuracy of EOB-MRI for the diagnosis of early HCC is approximately 95–100% [53]. One third of hypovascular hypointense nodules in hepatospecific phase become hypervascular ‘progressed’ HCC, with a 1 and 3-year. Therefore, the authors suggested that these hypovascular nodules should be strictly followed up or definitely treated as typical HCC [53]. In the study by Ahn et al. [54], 9 out of 84 HCCs (10.7%) were exclusively identified by hepatospecific phase and three were early HCCs, while in Golfieri et al. [55] 19 out of 20 early HCC remained unclassified at dynamic MRI alone because of atypical behavior and were diagnosed only in the hepatospecific phase. Golfieri et al. [56], in a pilot study, suggested that in atypical cirrhotic nodule, hypointensity in the hepatospecific phase is the most relevant diagnostic sign for differentiating low-risk from high-risk nodules, since the reduction of Gd-EOB-DTPA uptake seems to occur at an early stage of hepatocarcinogenesis which precedes the reduction of portal blood flow and nodule arterialization [56]. In fact an experimental study showed a gradual loss of the ability of hepatocytes to take up Gd-EOB-DTPA during hepatocarcinogenesis, according to the progression from dysplastic nodules to poorly differentiated HCC [57]. However, several authors documented that 5–10% of human HCCs can show a paradoxical uptake of Gd-EOB-DTPA in the hepatobiliary phase, appearing as iso- or hyperintense, whereas some dysplastic nodules can exhibit hypointensity [58–61]. However, according to Golfieri et al. [56], for atypical HCC hepatospecific phase hypointensity should be used as the second marker of malignancy.

### T2-W Hyperintensity

**Fig. 5:**
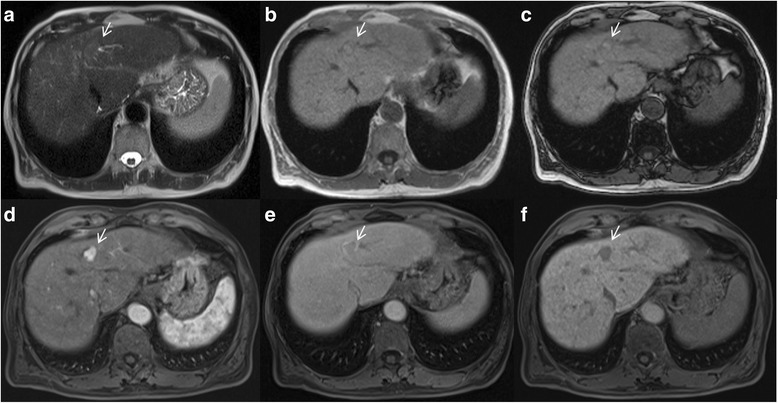
Man 74 years old with HCC on II hepatic segment. The HCC is hyperintense (*arrow*) on T2-W sequence (**a**), isointense (*arrow*) on T1-W (**b**: in-of-phase) with peripheral fat suppression (*arrow*) on T1-out of phase (**c**). During arterial phase of contrast study (**d**) with EOB-GD-DTPA, the HCC shows hyperenhancement (*arrow*), with wash-out and capsule appearance (*arrow*) on portal phase (**e**). During hepatospecific phase (**f**) of contrast study the HCC is hypointense (*arrow*)

According to LI-RADS, T2-W hyperintensity is an ancillary imaging features (Fig. 5). Park et al. [17] showed that dysplastic nodules and HCCs cannot be distinguished on the basis of signal intensity characteristics on unenhanced MRI, since their signal intensities are similar on T1- and T2-W sequences. However, dysplastic nodules are almost never hyperintense on T2-W, early HCCs are mostly isointense on T2-W, while higher grade (moderately or poorly) of HCC is associated with high signal intensity on T2-W images, although the signal intensity may also be related with tumor vascularity and peliotic changes [17]. Previous study demonstrated that T2-W hyperintensity was a highly specific marker of nodule malignancy, although poorly sensitive [27–62]. Golfieri et al. [56] showed that, compared to hypointensy on hepatospecific phase, T2-W hyperintensity was a poor predictor of malignancy in the early stages of HCC. Conversely to Golfieri [56], Ouedraogo et al. [63] demonstrated that the addition of T2-W hyperintensity to the AASLD criteria increased the detection rate of HCC, especially nodules smaller than 20 mm. In fact the sensitivity of MRI increased from 67.6 to 79%. Sofue et al. [64] evaluated the imaging features in MRI that are associated with upgrade of LI-RADS category observations to category 5, and demonstrated that the risk factors in the 56 LR-4 observations that upgraded to LR-5 were mild-moderate T2 hyperintensity (*P* < 0.001; hazard ratio = 1.84) and growth (*P* < 0.001; hazard ratio = 3.71). Although mild-moderate T2 hyperintensity was the most useful risk factor for predicting upgrade, actual risk level was only mildly elevated. Hwang et al. [65] compared the diagnostic performance of DWI and T2-W images, in differentiating between hypovascular HCC and dysplastic nodules seen as hypointense nodules at hepatobiliary phase. They showed that hyperintensity on T2-W and DWI were significant features for differentiating hypovascular HCCs from dysplastic nodules (*P* < 0.05), while there was no significant difference in mean ADC between hypovascular HCCs (1.06 ± 0.13) and dysplastic nodules (1.09 ± 0.13). The sensitivity of DWI was higher than T2-W (72.0% [18 of 25] versus 40.0% [10 of 25]). Hyperintensity on T2-W and DWI could be a useful imaging tool to differentiate hypovascular HCCs from dysplastic nodules seen as hypointense nodules in the hepatobiliary phase. Kim et al. [66] evaluated the most predictive finding among hyperintensity on T2-W, DWI, washout, capsular enhancement, and hypointensity on gadoxetic acid-enhanced hepatobiliary phase images in the detailed characterization of arterial phase enhancing nodules 1 cm in diameter and smaller. They showed that for hypervascular lesions 1 cm in diameter or smaller, T2-weighted images have the highest sensitivity among tests with an odds ratio statistically separable from 1 for differentiating HCC from benign hypervascular lesions 1 cm or smaller. Conversely Hussain et al. [67] concluded that T2-W images do not provide added diagnostic value in the detection and characterization of focal lesions because the heterogeneity and hyperintense fibrotic septa in the cirrhotic liver parenchyma can obscure moderately hyperintense HCC on T2-W images and that 42–53% of HCCs may be isointense to hypointense on T2-weighted images. Other researches also have reported that the combined use of hyperintensity on T2-W images improves differentiation of small non solid benign lesions from solid malignant tumors in the liver [68, 69].

### Restricted diffusion

**Fig. 6:**
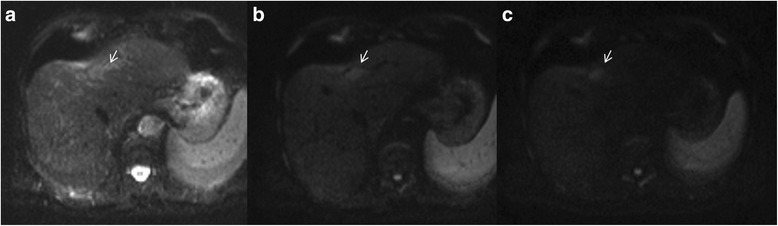
The same patient of Fig. 5. Restricted diffusion. The nodule (arrow) shows hyperintense signal on b0 s/mm^2^ (**a**), on b 500 s/mm^2^ (**b**) and on b 800 s/mm^2^(**c**)

The role of DWI in HCC patient has been evaluated by different studies [70–76]. Lee et al. [72] demonstrated that the addition of DWI to the gadoxetic acid-enhanced MRI could be a guideline in differentiating between HCCs and dysplastic nodules. In their study, 86 HCCs (84.3%) showed hyperintensity on DWI, whereas only three dysplastic nodules (13.0%) showed this feature. So they concluded that hyperintensity on DWI was highly indicative of HCC in patients with chronic hepatitis or cirrhosis. Also Piana et al. [73] showed that enhancement in the arterial phase and hyperintensity on DWI were found to be significantly more sensitive criteria for HCC than conventional criteria (77–76 vs. 60% for all HCCs and 66–60 vs. 37% for HCCs smaller than 20 mm). Sensitivity was even higher when enhancement in the arterial-dominant phase and washout (in the portal venous and/or equilibrium phases) or hyperintensity on DWI was used (84–85% for all HCCs and 71–74% for HCCs smaller than 20 mm). Granata et al. [74] demonstrated that that DWI could be used to predict the histological grade of HCC; in fact they found that there was a good correlation between ADC and grading, between perfusion fraction (fp) and grading, and between tissue pure diffusivity (Dt) and grading. Nakanishi et al. [75] showed not only the usefulness of DWI for histological grading, but also the possibility to use ADC as a preoperative prediction of early HCC recurrence within 6 months of operation. Conversely, Nasu et al., in a series of 125 resected HCCs (sizes range: 0.8–15 cm), found no correlation between histological grade and ADC (using b factors of 0 and 500 s/mm^2^), although the DWI and Signal Intensity of the HCCs increased in higher grade [76]. Sutherland et al. [77] compared ultrasound screening with DWI for detecting HCC. The sensitivity, specificity, positive predictive value and negative predictive values for US were 100, 90, 23 and 100%, respectively, while for MRI were 83, 98, 63 and 99%. The major advantage of DWI over US screening in this study has been the low false-positive rate of DWI. In fact US had false-positive studies 20 times (10%) while DWI had three false-positive examinations (2%). The reasons for the low false-positive rate of DWI include: not depicting macro regenerative and low grade dysplastic nodules and not depicting focal fatty heterogeneity, also the ability to correctly classify benign nodules as cavernous haemangiomas which usually have elevated apparent diffusion coefficient (ADC) values. They concluded that more studies are needed to validate the DWI as a screening tool and therefore it should replace US as a cost-effective screening tool [77]. DWI could be used as a helpful diagnostic tool for HCC in patients with chronic liver disease, since DWI can accurately detect HCC in patients with chronic liver disease regardless of the lesion size (Fig. 6). A potential reason for the better accuracy of DWI is that this does not rely on morphologic features only. Malignant tissues tend to be hypercellular with an accumulation of macromolecular proteins leaving a small extracellular space resulting in a decrease of the ADC value [78]. The major limits of DWI are the different parameters used in DWI sequences which may affect the results of ADC calculation. The different b values, selection method, bias of patient selection, pathological characteristic of lesions and measurement of ADC values may be reduced the reproducibility of the data, however all analyzed studies showed that the mean ADC value of malignant lesions was lower than that of benign lesions [70–80].

### Other ancillary features (intalesional fat, corona enhancement, mosaic architecture and iron sparing in iron overloaded)

MR imaging diagnosis of HCC is based mainly on assessment of vascularity, capsule appearance, and signal intensity in the hepatobiliary phase. MR imaging also permit assessment of ancillary imaging features, that can be divided into those that favor the diagnosis of HCC specifically (intralesional fat, corona enhancement, nodule-in-nodule architecture, and mosaic architecture) and those that favor the diagnosis of malignancy but are not specific for HCC (mild-moderate T2 hyperintensity, restricted diffusion, and lesional iron sparing) [5–7, 81].

Intralesional fat is the presence of lipid within a nodule in higher concentration than in the hepatic parenchyma [6]. This feature can be detected at MR by observing signal loss on out-of-phase compared with in-phase T1-weighted GRE images. In a patient at risk for HCC, the detection of intralesional fat in a solid nodule raises concern for malignancy or premalignancy. In fact, this feature does not establish the diagnosis of HCC, however, as the differential diagnosis includes high-grade dysplastic nodule and occasionally low-grade dysplastic nodule [82].

Corona enhancement is a feature of hypervascular, progressed HCC and refers to enhancement of the venous drainage area in the peritumoral parenchyma [16]. It is as a rim (“corona”) of enhancement around a progressed, hypervascular HCC in the late arterial phase or early portal venous phase, with fading to isoenhancement at subsequent phases. This feature begins a few seconds after tumor enhancement, so that corona and tumor enhancement may appear to overlap. This overlap may cause the tumor to appear larger than it really is. Its presence helps to differentiate small hypervascular HCCs from pseudolesions, however it is not a feature of early HCC [16, 82].

Mosaic architecture refers to the presence within a mass of randomly distributed internal nodules differing in enhancement, intensity, often separated by fibrous septa. This feature is characteristic of large HCCs and reflects the mosaic configuration observed at pathologic evaluation. It is unusual in tumors other than HCC [82].

Lesional iron sparing refers to relative paucity of iron in a solid mass compared with that of background iron-overloaded liver. This feature raises concern for premalignancy or malignancy because high-grade dysplastic nodules and HCCs characteristically are iron “resistant”. However it is not specific for high-grade dysplastic nodule or HCC, but other non-HCC malignancies may have this appearance [82].

## Conclusion

Early diagnosis is a critical step in the management of HCC patients. The identification of the specific vascular profile characterized by contrast arterial uptake followed by washout in the venous phases has 100% specificity when demonstrated on dynamic contrast study, in patients at high risk of HCC. Although the arterial phase hyperenhancement is an essential prerequisite for definitely HCC, it is not sufficient for LR-5 categorization. Hypointensity on hepatospecific phase and wash-out appearance are the most relevant diagnostic sign for differentiating low-risk from high-risk nodules in patients at risk for HCC. Therefore the use of EOB-GD-DTPA should be considered in this category of patients. The capsule appearance, T2-W hyperintensity and restricted diffusion have a high positive predictive value for HCC and may be associated to other imaging features for LIRADS characterization.
